# Lessons from *Mycobacterium avium* complex-associated pneumonitis: a case report

**DOI:** 10.1186/1752-1947-2-152

**Published:** 2008-05-13

**Authors:** Victor Zota, Sheryn M Angelis, Armando E Fraire, Ciaran McNamee, Shasta Kielbasa, Daniel H Libraty

**Affiliations:** 1 Department of Pathology, Division of Infectious Disease, University of Massachusetts Medical School, Worcester, MA, USA; 2 Department of Medicine, University of Massachusetts Medical School, Worcester, MA, USA; 3 Department of Surgery, University of Massachusetts Medical School, Worcester, MA, USA; 4 Center for Infectious Disease and Vaccine Research, University of Massachusetts Medical School, Worcester, MA, USA

## Abstract

**Introduction:**

*Mycobacterium avium *complex (MAC) is an increasingly recognized cause of pulmonary disease in immunocompetent individuals. An acute form of MAC lung disease, MAC-associated pneumonitis, has generally been associated with the use of hot tubs. There is controversy in the literature about whether MAC-associated pneumonitis is a classic hypersensitivity pneumonitis or is a direct manifestation of mycobacterial infection.

**Case presentation:**

We report the second case in the literature of MAC-associated pneumonitis not related to the use of hot tubs. The source of MAC in a 52-year-old immunocompetent patient was an intrapulmonary cyst containing numerous acid-fast bacilli. The patient developed disseminated miliary nodules throughout both lung fields. Histological examination of resected lung tissue revealed well-formed, acid-fast negative granulomas composed predominantly of CD4^+ ^T-cells and CD68^+ ^histiocytes. The granulomas were strongly positive for tumor necrosis factor-α, a pro-inflammatory cytokine.

**Conclusion:**

The attempt to classify MAC-associated pneumonitis as either a classic hypersensitivity pneumonitis or a direct manifestation of mycobacterial infection is not particularly useful. Our case demonstrates that MAC-associated pneumonitis is characterized by a vigorous T-helper 1-like, pro-inflammatory, immune response to pulmonary mycobacterial infection. The immunopathology provides a rationale for clinical studies of anti-MAC therapy with the addition of anti-inflammatory agents (for example, corticosteroids) to hasten the resolution of infection and symptoms.

## Introduction

*Mycobacterium avium *complex (MAC)-associated pneumonitis has typically been reported in immunocompetent persons exposed to aerosolized MAC from indoor or outdoor hot tubs (termed "hot tub lung") [1-3]. There is controversy in the literature about whether the illness is a classic hypersensitivity pneumonitis or is a direct manifestation of mycobacterial infection. Histopathologic and bronchoscopic differences have been described between classic hypersensitivity pneumonitis and MAC-associated pneumonitis [2,4].

We present here the second reported case of MAC-associated pneumonitis that was not associated with the use of hot tubs [2]. The pathological features of this case of MAC-associated hypersensitivity pneumonitis stress the differences between 'classic' hypersensitivity pneumonitis and MAC-associated pneumonitis. These differences have important implications for the diagnosis as well as for the treatment of MAC-associated pneumonitis. Our report also highlights the clinical importance of alternative sources of MAC that can lead to a hypersensitivity pneumonitis.

## Case presentation

**Figure 1:**
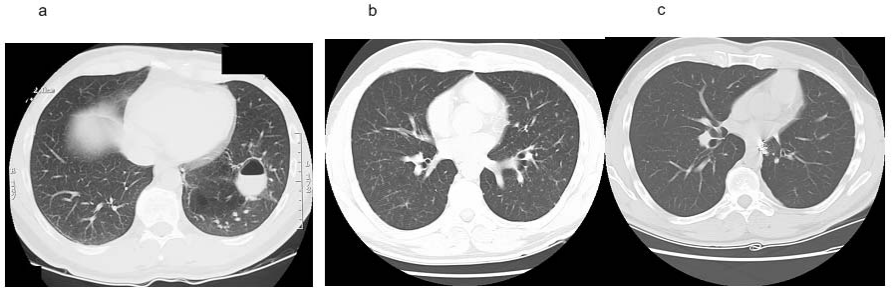
**HRCT appearance of left lower lobe cyst, MAC-associated pneumonitis, and subsequent resolution**. **(a) **Left lower lobe cyst with air-fluid level and adjacent infiltrate at initial presentation. **(b) **Bilateral, diffuse miliary nodular pattern six months later. **(c) **Resolution of miliary pattern after 3 months of antimycobacterial treatment.

A 52-year-old man presented with two weeks of dyspnea and a productive cough. A high-resolution computed tomography (HRCT) scan of the chest showed a left lower lobe cyst with an air-fluid level and adjacent infiltrate (Figure 1a). He was treated with levofloxacin, which resolved the symptoms. Six months later he began to experience fatigue, dyspnea and a productive cough. A chest radiograph showed complete opacification of the cyst in the left lower lobe. He was treated empirically with azithromycin and levofloxacin, without improvement. A bronchoscopy with bronchoalveolar lavage was unrevealing. Routine bacterial, acid-fast bacilli (AFB), and fungal stains and cultures were negative. After four weeks, the patient continued to have a productive cough and fatigue, worsening dyspnea, weight loss and fevers. He was admitted to the hospital for resection of a presumed non-resolving lung abscess.

His past medical history was unremarkable. He did not take any immunosuppressive medications and had no exposure to hot tubs. Physical examination revealed mild hypoxemia and coarse rales over the left lower lung field. White blood cell count was 12,400/mm^3^. HIV serology was negative, as was a tuberculin skin test. Blood and urine cultures were negative. A pre-operative HRCT showed a new diffuse pattern of miliary nodules throughout both lung fields (Figure 1b). No ground glass opacities were seen. Because of the central location of the lesion a left lower lobectomy was performed. Examination of the resected lobe revealed a cyst filled with thick yellow fluid that was AFB positive on a smear. Cultures from the cyst fluid eventually grew MAC; bacterial and fungal cultures were negative.

The patient was initially placed on a four-drug anti-tuberculosis regimen. After identification of MAC in the culture, his antibiotic regimen was changed to azithromycin, ethambutol, and rifampin. His respiratory symptoms resolved over the ensuing three months, with disappearance of the miliary nodules on chest HRCT (Figure 1c).

**Figure 2:**
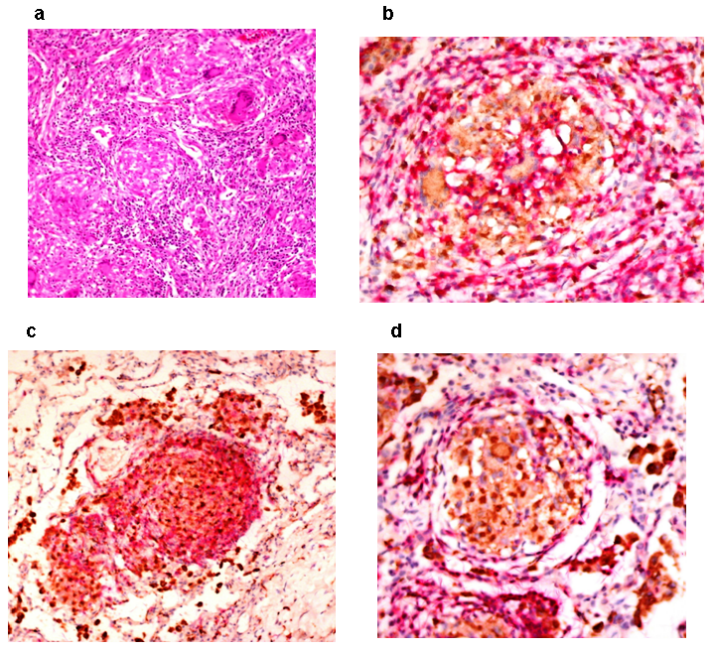
**Immunohistochemical staining for macrophages (CD68) and T-lymphocyte subsets (CD5, CD4, and CD8) in lung tissue biopsy**. **(a) **Lung tissue with well-formed granulomas, hematoxylin and eosin stain, 200× magnification. **(b) **Anti-CD68 (brown) and anti-CD5 (red) immunostaining, 400× magnification. **(c) **Anti-CD68 (brown) and anti-CD4 (red) immunostaining, 200× magnification. **(d) **Anti-CD68 (brown) and anti-CD8 (red) immunostaining, 40× magnification.

**Figure 3:**
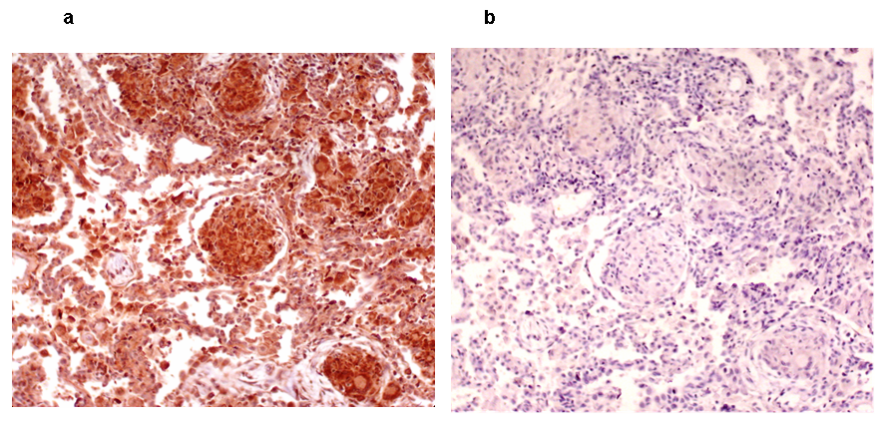
**Immunohistochemical staining for tumor necrosis factor-α in lung tissue biopsy**. **(a) **Anti-tumor necrosis factor-α immunostaining (brown), 200× magnification. **(b) **Isotype control antibody immunostaining (negative control) for anti-tumor necrosis factor-α, 200× magnification.

Histologic examination of the resected lung lobe revealed a bronchogenic cyst. The lung parenchyma surrounding the cyst was replaced by well-formed granulomas that were AFB negative (Figure 2a). The granulomas consisted of central CD68^+ ^macrophages surrounded by a mantle of CD5^+ ^T-lymphocytes, with some T-lymphocytes infiltrating the granulomas (Figure 2b). CD4^+ ^T-cells constituted the main lymphocytic component of the granulomas and were closely associated with the CD68^+ ^macrophages in the central core (Figure 2c). CD8^+ ^T-cells were essentially restricted to the periphery, and there was a paucity of CD20^+ ^B-cells in the perigranulomatous areas (Figure 2d). The central core of the granulomas and the surrounding tissue showed strong reactivity for tumor necrosis factor-α (TNF-α) (Figure 3).

## Discussion

MAC infection activated a vigorous immune response in our patient that, together with the antimycobacterial regimen, likely contributed to the resolution of disease. The antimycobacterial immune response was characterized by well-formed granulomas with a low organism load, increased CD4/CD8 ratio in the granulomas, and marked TNF-α production. This T-helper 1-like immune response is considered pivotal for the eradication of mycobacterial pathogens. It is also somewhat different than what is reported in classic hypersensitivity pneumonitis. The histologic appearance of classic hypersensitivity pneumonitis is characterized by poorly formed, non-necrotizing granulomas, and patchy interstitial pneumonitis. It is described as a combination of IgG-mediated and CD8^+ ^> CD4^+ ^T-cell mediated hypersensitivity reactions [5]. One possible exception is farmer's lung, which is often characterized by increased CD4/CD8 ratios [6].

In our patient, the TNF-α+ CD4^+ ^T-cell dominant granulomatous immune response produced clinical symptoms and diffuse miliary nodules. The MAC organism load was high in the cyst fluid, but low in the diffuse parenchymal granulomas. This may suggest that heavy or continuous exposure to MAC (for example, in hot tubs or, in this case, from a heavily colonized cyst) promotes the development of antimycobacterial T-helper 1-like immune responses in the lung. This case is also a reminder that pulmonary structural abnormalities may be a reservoir for triggering MAC-associated pneumonitis.

## Conclusion

The attempt to classify MAC-associated pneumonitis as either a classic hypersensitivity pneumonitis or a direct manifestation of mycobacterial infection is not particularly useful. Our case demonstrates that MAC-associated pneumonitis is caused by a vigorous T-helper 1-like immune response to pulmonary MAC infection. The T-helper 1-like antimycobacterial immune response is consistent with the observation that MAC-associated pneumonitis is generally a self-limited disease [1]. Corticosteroids were not used in this case. However, the observed immunopathology provides a rationale for clinical studies of the use of anti-MAC therapy and anti-inflammatory agents (for example, corticosteroids) to hasten the resolution of infection and symptoms.

## Abbreviations

AFB, acid-fast bacilli; HRCT, high-resolution computed tomography; MAC, *Mycobacterium avium *complex; TNF-α, tumor necrosis factor-α.

## Competing interests

The authors declare that they have no competing interests.

## Authors' contributions

VZ and SMA contributed to the content and drafting of the manuscript. AEF facilitated and interpreted the immunohistologic staining of the lung tissue and contributed to the drafting of the manuscript. CMcN participated in the care of the patient and provided the tissue specimens. SK performed the TNF-α immunostaining. DHL conceived of the study, participated in the care of the patient, and participated in all other aspects noted above. All authors read and approved the final manuscript.

## Consent

Written informed consent was obtained from the patient for publication of this case report and any accompanying images. A copy of the written consent is available for review by the Editor-in-Chief of this journal.
